# The daily practice reality of PD-L1 (CD274) evaluation in non-small cell lung cancer: A retrospective study

**DOI:** 10.3892/ol.2020.11458

**Published:** 2020-03-12

**Authors:** Camille Verocq, Christine Decaestecker, Laureen Rocq, Sarah De Clercq, Audrey Verrellen, Zita Mekinda, Sebahat Ocak, Christophe Compère, Claudia Stanciu-Pop, Isabelle Salmon, Myriam Remmelink, Nicky D'Haene

**Affiliations:** 1 Department of Pathology, Erasme Hospital, Université Libre de Bruxelles (ULB), 1070 Brussels, Belgium; 2 DIAPath-Center for Microscopy and Molecular Imaging, ULB, 6041 Gosselies, Belgium; 3 Laboratory of Image Synthesis and Analysis, Ecole Polytechnique de Bruxelles, ULB, 1050 Brussels, Belgium; 4 Department of Pulmonology, Erasme Hospital, ULB, 1070 Brussels, Belgium; 5 Division of Pulmonology, Centre Hospitalier Universitaire (CHU) Université Catholique de Louvain (UCL) Namur (Godinne Site), UCL, 5530 Yvoir, Belgium; 6 Pole of Pulmonology, Institut de Recherche Expérimentale et Clinique, UCL, 1200 Brussels, Belgium; 7 Department of Pulmonology, Centre Hospitalier Inter Régional Edith Cavell Cancer Institute, 1160 Brussels, Belgium; 8 Department of Pathology, CHU UCL Namur (Godinne Site), UCL, 5530 Yvoir, Belgium; 9 Centre Universitaire Inter Regional d'Expertise en Anatomie Pathologique Hospitalière, 6040 Charleroi, Belgium

**Keywords:** CD274 molecule, PD-L1, IHC, NSCLC, daily practice, concordance

## Abstract

Treatment with pembrolizumab, an anti-programmed cell death-1 (PDCD-1) monoclonal antibody for the treatment of non-small cell lung cancers (NSCLCs) requires prior immunohistochemical (IHC) analysis of the expression of the programmed death-ligand 1 (PD-L1) (also known as CD274 molecule) which is a heterogeneous and complex marker. The present study aimed to investigate how pathological and technical factors (such as tumor location and sampling type, respectively) may affect the PD-L1 evaluation in patients with NSCLC in the daily practice of pathology laboratories. The current study was retrospective, and included 454 patients with NSCLC, for whom PD-L1 expression analysis by IHC was prospectively performed between November 2016 and January 2018. The association between PD-L1 expression and the clinicopathological characteristics of patients was statistically investigated using either the χ^2^ and Fisher exact tests or the Mann-Whitney and Kruskal-Wallis tests, depending on whether PD-L1 expression was assessed in three large categories (<1, 1–49, ≥50%) or in more precise percentages. Furthermore, the same statistical methodology was used to analyze the heterogeneity of PD-L1 expression according to its sampling type (cytology, biopsy or surgical specimen) and its location (primary tumor, lymph node or distant metastasis). Intra- and inter-observer discrepancies were also studied using double-blind evaluation and concordance analyses based on the weighted κ coefficient. The results demonstrated a significant association between PD-L1 expression and sample location (P=0.005), histological type (P=0.026), total number of mutations (P=0.004) and KRAS proto-oncogene, GTPase mutations (P=0.024). In addition, sampling type did not influence PD-L1 expression. The inter- and intra-observer discrepancies were 15% and between 16 and 17.5%, respectively. The present study confirmed that evaluation of PD-L1 expression by IHC can be performed on all types of samples. In addition, the results from the current study highlighted the heterogeneity of PD-L1 expression among the different types of sample location. In complex cases, a second evaluation of PD-L1 expression by IHC would be performed due to intra- and inter-observer discrepancies.

## Introduction

In July 2015, the European Medicines Agency approved the use of immunotherapy (IT) targeting the programmed cell death 1 (PDCD-1)/programmed death-ligand 1 (PD-L1) (also known as CD274 molecule) interaction in patients with locally advanced, non-resectable or metastatic non-small cell lung cancers (NSCLCs) (1,2). However, since the response rate of NSCLC to IT varies from 12 to 49%, it is crucial to determine biomarkers to identify responders and non-responders (1).

Numerous studies have reported the use of PD-L1 expression evaluation by immunohistochemistry (IHC) as a biomarker that could predict the effectiveness of anti-PDCD-1/PD-L1 IT, in order to select patients who would benefit from this type of therapy (3–5). The Checkmate 057 (6), Keynote 001 (7) and POPLAR (8) clinical studies demonstrated that, in NSCLC, high expression of PD-L1 in tumor cells is associated with a better response to IT; however the Checkmate 017 (9) study reported that some patients responded positively to nivolumab, another IT drug, although their lung tumor did not express PD-L1. At present, only the use of pembrolizumab is based on PD-L1 expression (1,10,11).

The contradictory results regarding the theranostic role of PD-L1 expression can be explained by several parameters. Firstly, PD-L1 expression is heterogeneous and dynamic. It varies between the primary tumor and metastasis, and within the tumor itself (4,12). Hendry *et al* (13) analyzed PD-L1 expression in numerous samples from the same tumor with a low to moderate agreement (13). This heterogeneity partly explains the differences in PD-L1 expression observed by Ilie *et al* (14) in biopsies compared with that in surgical resections, with a lower PD-L1 expression in biopsies. Conversely, previous studies reported a good agreement between histological and cytological samples, which are widely used in clinical routines (10,15,16). Cytological sample analysis could therefore be recommended routinely, although its use has not yet been validated in clinical trials (11). Secondly, it should be noted that for each IT molecule, a specific test for the evaluation of PD-L1 expression is developed (1). These tests involve different antibodies and platforms that make cross-comparisons difficult. However, numerous studies have reported encouraging results (10,17–19). For example, the Blueprint Phase 1 (20) and the French Harmonization studies (21) reported a good agreement for 28–8, 22C3 and SP263 antibodies, suggesting they can be potentially interchangeable, whereas the SP142 assay exhibited fewer stained tumor cells (7,13). The Blueprint Phase 2 study which used clinical routine samples confirmed the lower sensitivity of the SP142 assay and reported a higher sensitivity with the 73–10 antibody (16). Thirdly, interpretation of PD-L1 expression by IHC can be difficult. PD-L1 is expressed by immune, tumor and necrotic cells in the membrane or cytoplasm, whereas the theranostic criterion only considers the membrane staining of viable tumor cells (1). These difficulties can negatively impact the intra- and inter-observer concordance regarding the PD-L1 assessment, which are the levels of agreement respectively when the same pathologist assesses the slides twice and when two different pathologists assess the same slides double-blinded (10,17,22–24). In all cases, the intra-observer agreement increases with the PD-L1 expression (10).

The aforementioned data were obtained from retrospective and prospective clinical studies conducted on homogeneous series, according to numerous selection criteria. Apart from the Blueprint Phase 2, only a few studies were conducted on series extracted from clinical routine samples (25). Therefore the influence of the complexity (in regards to the biology, technicality or interpretation) of PD-L1 analysis on the daily management of patients was evaluated. The present retrospective study analyzed the impact of pathological and technological factors on the daily evaluation of PD-L1 in patients with NSCLC. Nowadays molecular tumor profiling represents an integral part of pathologist's daily practice for patients with NSCLC and allows personalized medicine. At Erasme Hospital (Brussels, Belgium), NSCLCs are daily characterized using a next generation sequencing (NGS)-based gene panel targeting 22 genes (AKT1, ALK, BRAF, CTNNB1, DDR2, EGFR, ERBB2, ERBB4, FBXW7, FGFR1, FGFR2, FGFR3, KRAS, MAP2K1, MET, NOTCH1, NRAS, PIK3CA, PTEN, SMAD4, STK11 and TP53). These data were therefore included in the present study, and variations in PD-L1 expression according to molecular (NGS) data were analyzed.

## Materials and methods

### 

#### Clinical series

The present retrospective study included 454 patients with NSCLC for whom the PD-L1 status was requested by clinicians, and was approved by the Ethics Committee of Erasme Hospital (Brussels, Belgium; approval no. P2017/581). The Ethics Committee waived the need for written informed consent from all participants. Between November 2016 and January 2018, a total of 454 formalin-fixed paraffin-embedded (FFPE) samples obtained from patients with NSCLC following biopsy and/or surgical resection were received from 12 different institutions and used for the analysis of PD-L1 expression by IHC (on 4-µm thick tissue sections). The FFPE blocks were provided by the Pathology Department of Erasme Hospital (Brussels, Belgium), the Centre Universitaire Inter Regional d'Expertise en Anatomie Pathologique Hospitalière (CurePath, Charleroi, Belgium), the Centre Hospitalier Universitaire (CHU) Université Catholique de Louvain (UCL) Namur (Godinne Site, Belgium), the Centre de Morphologie Pathologique (CMP; Brussels, Belgium), the Institut Jules Bordet (Brussels, Belgium), the CHU Charleroi (Belgium), the CHU Brugmann (Brussels, Belgium), the Centre Hospitalier de Mouscron (Belgium), the Cliniques du Sud Luxembourg (Edmond-Jacques Site, Belgium), the CHR Verviers (Belgium), the Centre Hospitalier EpiCURA (Frameries Site, Belgium), and the Centre Hospitalier de Wallonie picarde (CHwapi, Union Site, Belgium). PD-L1 expression analysis by IHC was performed at the Pathology Department of Erasme Hospital. To respond to recurrent clinician requests, among the 454 patients we included 87 patients for whom only cytological samples were available (including endobronchial ultrasound-guided transbronchial needle aspiration, pleural effusion and bronchial aspiration). In the present study, the sampling type (cytology, biopsy or surgery) and the sample location (primary tumor, LN or distant metastasis) were distinguished. The pathological tumor (pT), node (pN), metastasis (pM) and stage were revised according to the 8th UICC edition (26). All samples included in the present study met the acceptability criterion of ≥100 viable (non-necrotic) tumor cells that was required for the companion test used (Dako PharmDx 22C3; Dako; Agilent Technologies, Inc.).

Test requests from external centres were reviewed and, when provided, clinical, histopathological, IHC and molecular data were extracted. Moreover, for cases from Erasme Hospital, clinical, histopathological, IHC and molecular data were extracted from the medical records. Tables I and II present the clinical, histopathological, immunohistochemical and molecular data so collected for the patients included in the present study. As some information was missing, the total number of data available varies between the different features. As mentioned above, the molecular data were already available in the medical records and were the result of targeted NGS using the Colon and Lung AmpliSeq panel (Thermo Fisher Scientific, Inc.), as previously described (27).

#### IHC

IHC staining for PD-L1 was performed using PD-L1 IHC 22C3 pharmDx kit (cat. no. SK006; Dako; Agilent Technologies, Inc.) on the Autostainer Link 48 system (Dako; Agilent Technologies, Inc.) according to the manufacturer's instructions. Briefly, FFPE samples were cut into 4-µm thick sections. Deparaffinization, Rehydration and Target Retrieval (3-in-1) Procedure (reagents included in the PD-L1 IHC 22C3 pharmDx kit) was performed using PT Link Pre-treatment Module (Dako; Agilent Technologies, Inc.) according to the manufacturer's instructions. PD-L1 IHC was performed on the Autostainer Link 48 system (Dako; Agilent Technologies, Inc.) using the 22C3 antibody (monoclonal mouse anti-PD-L1 antibody; clone 22C3; ready-to-use; provided in the IHC 22C3 pharmDx kit). The sections were counterstained for 5 min with haematoxylin at room temperature. Quality controls were included in each staining run. Controls used FFPE tonsil samples in addition to PD-L1 IHC 22C3 pharmDx Slide of Control Cell Lines (provided in the IHC 22C3 pharmDx kit). Moreover, Negative Control Reagent (monoclonal mouse control IgG antibody; ready to use; provided in the IHC 22C3 pharmDx kit) was also used for each sample. IHC slides were analyzed using a bright field microscope (magnifications, ×2 to ×40; Olympus).

#### PD-L1 tumor proportion score (TPS) evaluation and heterogeneity analysis

The PD-L1 TPS was evaluated as the percentage of viable tumor cells presenting partial or complete PD-L1 expression at the cell membrane, as previously described (1). TPS was classified into three large categories, corresponding to <1, 1–49 or ≥50% of positively stained cells and labelled as such to facilitate understanding of the results. Depending of the type of statistical analysis, the ordinal property of these 3 categories was taken into account or not. In addition, TPS was also evaluated in 13 ordered and more specific categories, corresponding to 0, 1, 5, 10, 20, 30, 40, 50, 60, 70, 80, 90 and 100% of positively stained cells and labelled as such to facilitate understanding of the results. Subsequently, these 13 categories are referred to as ‘the precise TPS values’.

The heterogeneity of PD-L1 expression was evaluated in patients with multiple analysable samples. The PD-L1 TPS of these cases (n=80) was calculated by two pathologists [Termed pathologist 1 (junior) and pathologist 2 (senior)] who were double-blinded, allowing the assessment of intra- and inter-observer agreements. The first assessment for pathologist 2 was conducted prospectively (during the clinical routine) and the second retrospectively (i.e., all slides were reassessed together in a single evaluation session), whereas pathologist 1 performed each assessment retrospectively, with a wash-out period of two weeks between the two sessions. Regarding the analysis of inter-observer concordance, for each pathologist we used the average of their two precise TPS evaluations performed per case. Retrospective evaluation by a third pathologist (3, junior) was added to complete the inter-observer concordance analysis. For the analysis of the whole series, the PD-L1 values used correspond to the value retrieved from the pathological report and were considered as the first assessment by pathologist 2 in the concordance analysis.

For some patients (n=28), multiple samples from different locations were available and analysable (28 pairs, including 16 patients with primary tumor and LN samples and 12 patients with primary tumor and distant metastasis samples). The paired PD-L1 expression levels obtained were compared. Comparative analysis were also performed in 14 other patients, for whom two types of samples (surgical resection and either cytology or biopsy) were available.

#### Statistical analysis

The statistical analyses were performed using Statistica 7.1 software (StatSoft, Inc.). χ^2^ tests were used to analyze the associations between the PD-L1 TPS assessed in 3 categories (<1, 1–49 or ≥50%) and all the categorical clinicopathological variables described in Table I. In these analyses the ordinal property of the 3 TPS categories was not considered. Before applying the χ^2^ it was checked whether the expected frequencies (computed under the null hypothesis of independence) were non-zero and that the percentage of expected frequencies <5 was below 20%. In some case, categories were merged resulting in 2×2 contingency tables, on which we applied the Fisher's exact test. The variations of the PD-L1 TPS assessed in 13 ordered categories were also analyzed and considered as ranked data between two or more independent groups determined by clinicopathological variables, using Mann-Whitney tests or Kruskal-Wallis tests (with Dunn's multiple comparisons post hoc test using rank sums) respectively (see details in the results). The intra- and inter-observer concordance analyses were based on either the weighted κ coefficient (computed with an online calculator, ©Richard Lowry 2001–2019, http://vassarstats.net/kappa.html) to take into account the ordinal property of the TPS assessed in the three large categories, or Lin's concordance correlation coefficient (CCC) for TPS assessed by the means of the 13 more precise percentages that were considered as quantitative data (28).

## Results

### 

#### Factors impacting PD-L1 expression and its TPS value

**Figure 1.**
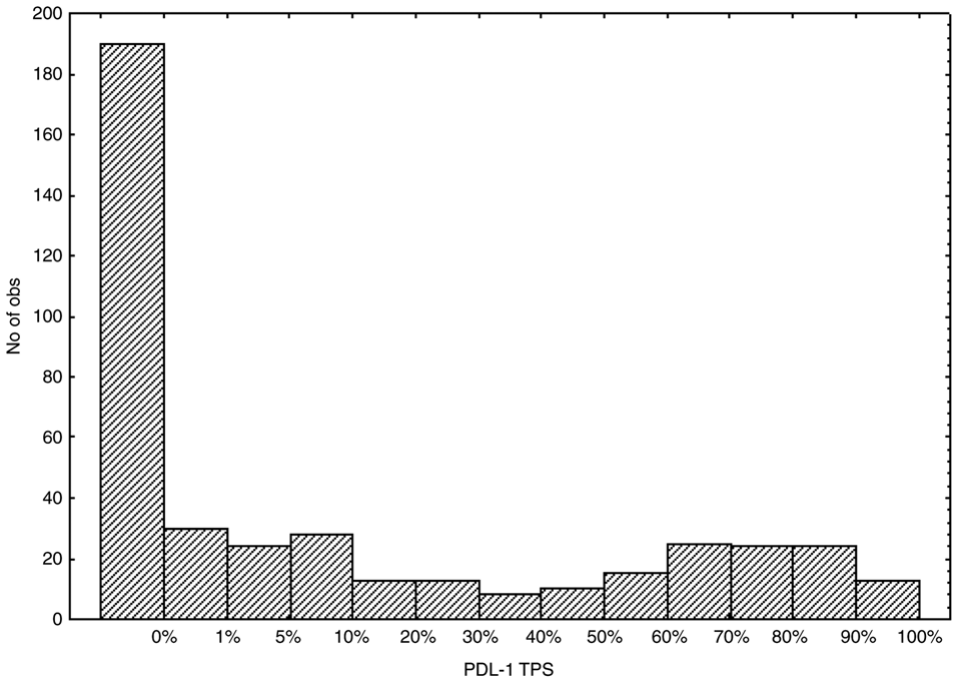
Distribution of PD-L1 TPS. Distribution of PD-L1 TPS values evaluated in 13 precise scores. No of obs: Number of observations; PD-L1, programmed death-ligand 1 molecule; TPS, Tumor Proportion Score.

The distribution of the PD-L1 TPS from the NSCLC samples according to the 13 categories is presented in Fig. 1. Following grouping of the TPS values in three large categories, 190 cases (41.85%), 133 cases (29.30%) and 131 cases (28.85%) had a TPS score corresponding to <1, 1–49 and ≥50% of positively stained cells, respectively.

Subsequently, whether and how the clinicopathological characteristics listed in Tables I and II affect the PD-L1 TPS evaluation was analyzed. The different methods used to score PD-L1 expression (using either three or 13 categories) were considered in the analysis. Only significant variations are summarized in Table III, including a refined analysis carried out on the positive cases (precisely TPS values ≥1%) only.

First, the sampling type (surgical resection, biopsy or cytology) and the age of the FFPE block had no significant impact on PD-L1 expression. The absence of variation between the different sampling types was confirmed in a small series of 14 patients for whom two types of samples were available (surgical resection and either cytology or biopsy). For these 14 sample pairs, the TPS evaluation in three categories perfectly matched, and the precise TPS evaluation agreed with a CCC of 0.965 and a 95% confidence interval (CI) of (0.895–0.989).

**Figure 2.**
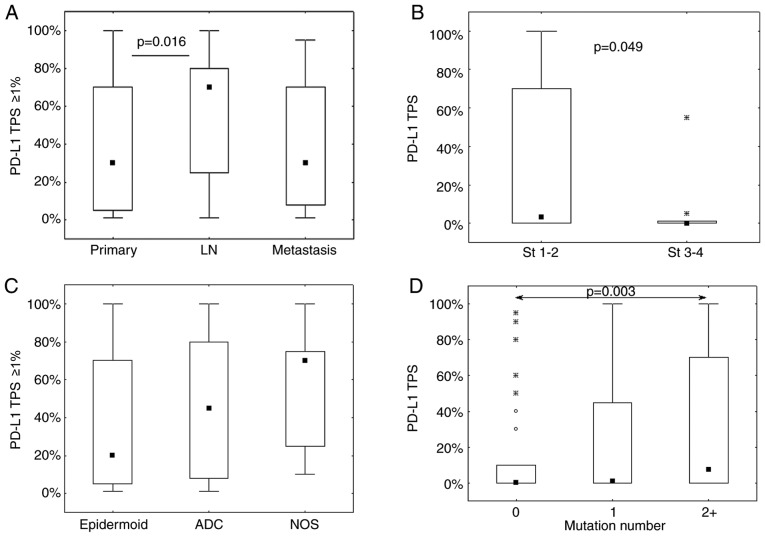
Factors impacting PD-L1 expression and its TPS value. PD-L1 TPS values according to (A) sample location, (B) clinical stage, (C) histological subtypes and (D) mutation number. Only significant pairwise differences were indicated (using Mann-Whitney test in B; Dunn's multiple comparisons post hoc test in A, C and D for which Kruskal-Wallis P-values are provided in Table III). ADC, adenocarcinoma; LN, Lymph Nodes Metastasis; NOS, non-otherwise specified; PD-L1, programmed death-ligand 1; St, clinical stage; TPS, Tumor Proportion Score.

**Figure 3.**
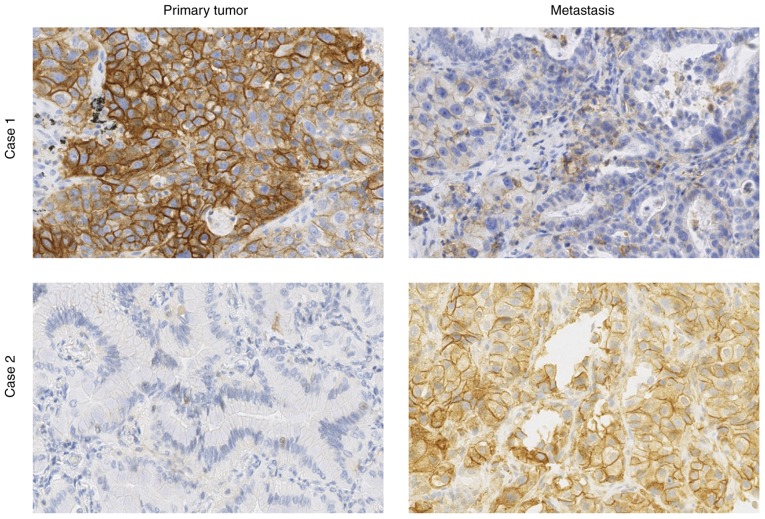
Heterogeneity of PD-L1 expression. PD-L1 staining patterns in pairs of primary tumor and metastasis samples from the same patient illustrating the heterogeneity of PD-L1 expression. Case 1, TPS=90% in tumor and 10% in metastasis. Case 2, TPS=0% in tumor and 90% in metastasis. Magnification, ×40. PD-L1, programmed death-ligand 1; TPS, Tumor Proportion Score.

Conversely, as detailed in the top of Table III, the sample location is significantly associated with the TPS assessed in 3 categories (P=0.005). TPS was >50% in 35% (23/66) of distant metastases and in 38% (33/88) of LN, whereas it was only observed in 24% (63/260) of primary tumors. In addition, LN exhibited the highest rate of PD-L1-negative cases (48% vs. 41 and 36% for primary tumor and distant metastases, respectively). However, in the positive cases (TPS ≥1%), a refined analysis on the precise TPS values demonstrated a significant TPS increase in LN compared with primary tumors (Fig. 2A). The analysis was completed by comparing pairs of samples from the same patient (from primary tumor and either LN or distant metastasis; Table IV and Fig. 3). The data was fairly heterogeneous from one patient to another, as shown in Fig. 3. Two very different PD-L1 staining profiles in primary tumor and metastasis from the same patient are presented (Fig. 3). In case 1 the expression was strongly higher in the primary tumor than in the metastasis, whereas the converse was true in case 2. It was observed that 43% of primary tumors not expressing PD-L1 had PD-L1-positive LN or metastasis, whereas 29% of PD-L1-positive primary tumors had PD-L1-negative LN or metastasis. The PD-L1 TPS categories were the same for only 11 of 28 pairs (see diagonal in Table IV), and precise TPS values were concordant for eight pairs only, with no significant concordance correlation (CCC, 0.189; CI, −0.166–0.501; data not shown). The observations from these paired samples demonstrated that most of the positive cases exhibited intermediate PD-L1 TPS (i.e. 1–49%) in LN/metastases vs. high TPS (i.e. ≥50%) in primary tumors, contrasting with the results obtained in the complete series (Table III).

Considering the variations observed with the sample location, the impact of stage and pT, pN, pM on PD-L1 expression were evaluated for the primary tumors only. Negative PD-L1 expression (TPS <1%) was observed in 55% of stages 3–4 and 29% of stages 1–2 (see Table III). Significant variations were evidenced between stages when considering precise TPS values (see Fig. 2B). No significant variations were observed with respect to the pTNM variables (data not shown).

Histology is also significantly associated with the TPS assessed in 3 categories (P=0.026, see Table III). PD-L1 expression was less often negative in SCC (37/112; 33%) than in ADC (129/290; 44%) and NOS histological subtypes (13/24; 54%). However, the positive PD-L1 TPS in SCC was more frequently between 1 and 49% compared with 50% or more, whereas the opposite trend was observed for the positive TPS values in ADC and NOS (Table III). These data were confirmed by the analysis of the precise positive PD-L1 scores (TPS ≥1%) that demonstrated a significant variation (with the same trend) among the three histological subgroups (P=0.036; Fig. 2C).

The results from targeted NGS demonstrated a significant association between the number of mutations and PD-L1 expression (Table III). It was observed that the absence of any mutations was associated with an absence of PD-L1 expression, whereas PD-L1 TPS increased with the mutation number (Table III and Fig. 2D). Regarding the specific gene mutations, no significant association was found between PD-L1 expression and epidermal growth factor receptor (*EGFR)* or tumor protein p53, with or without considering histological subtype stratification (data not shown). Conversely, we observed that the patients with NSCLC and presenting with *KRAS* mutations expressed PD-L1 more frequently (65% of TPS values ≥1%) compared with patients with NSCLC without *KRAS* mutations (47% of TPS values ≥1%), resulting in a significant association between the two categorical variables (P=0.024; Table III). A significant difference between the two *KRAS* mutation-related groups was also observed in terms of the precise evaluation of the PD-L1 TPS, which was higher in the mutated group (P=0.0006, data not shown). Furthermore, the available data showed that at least 80% of SCC and NSCLC NOS samples (i.e. 12 of 14 and 16 of 20 cases respectively) presented no *KRAS* mutations compared with only 60% (i.e. 142 of 236) for ADC samples (P=0.041). A specific analysis on patients with ADC only provided similar results to all patients, and demonstrated 62% (i.e. 55 of 89 cases) of positive PD-L1 expression in the presence of *KRAS* mutation vs. 45% (i.e. 59 of 132) in the absence of *KRAS* mutation (Fisher's exact test: P=0.014). The analysis of the three most frequent *KRAS* mutations (*G12C, G12V* and *G12D*) did not provide significant association with PD-L1 expression.

#### Intra- and inter-observer concordance analysis

The weighted κ index characterizing the intra-observer agreement of the PD-L1 TPS assessment in three categories was 0.779 (CI, 0.665–0.893) for pathologist 1 and 0.804 (CI, 0.703–0.905) for pathologist 2, with 17.5 and 16% of cases with discordances, respectively (Table V). For both observers, these discordances concerned the three sampling types (surgical resection, biopsy and cytology). Most discordances were found in the intermediate 1–49% category, where ~1/3 of the cases classified as intermediate (1–49%) at the first assessment were reclassified as negative (<1%) at the second assessment (Table V). The results also demonstrated that only four intra-observer discordances were common to both pathologists and concerned different types of samples (one surgical resection, one biopsy and two cytology samples). The CCC characterizing the intra-observer agreement of the PD-L1 TPS assessment in 13 precise values were 0.978 (CI, 0.959–0.988) for pathologist 1 and 0.946 (CI, 0.888–0.975) for pathologist 2.

**Figure 4.**
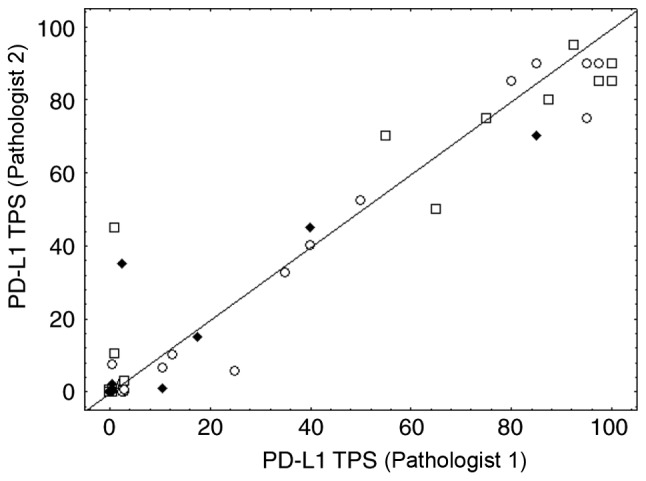
Inter-observer concordance. Inter-observer concordance for the precise PD-L1 TPS assessments between pathologist 1 (junior) and pathologist 2 (senior). Dots, squares and diamonds represent surgery, biopsy and cytological samples, respectively. Some pairs with small values resulted in many overlaps. The value pair (0,0) was observed 33 times, the pair (0,0.5) 4 times, (0.5,0) 5 times, the pair (0.5,0.5) for 2 times and the pair (3,0.5) for 2 times. PD-L1, PD-L1 molecule; TPS, tumor proportion score.

**Figure 5.**
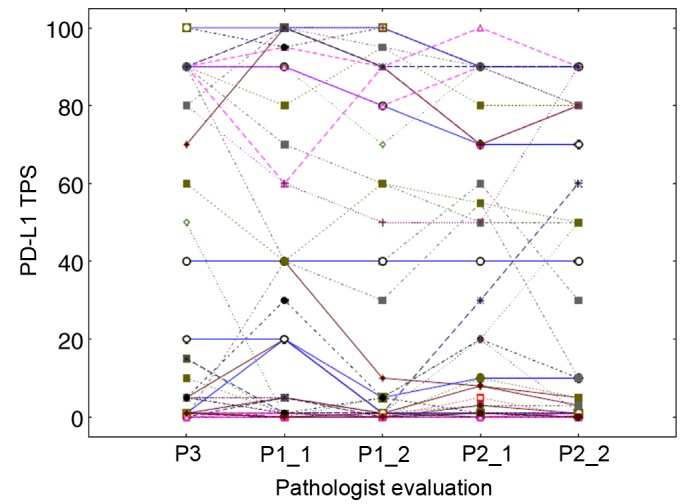
Overview of the intra- and inter-observer variations. The PD-L1 TPS assessed by pathologist 3 (junior, 1 evaluation), pathologist 1 (junior, 2 evaluations) and pathologist 2 (senior, 2 evaluations) were linked per case. Each case is identified by a line with a specific shape and colour and connecting all pathologists' assessments. A total of 24 perfect agreements of the 5 assessments on the TPS value of 0 were observed, resulting in overlapping horizontal lines at level 0. PD-L1, PD-L1 molecule; TPS, tumor proportion score; P, pathologist.

In order to evaluate the inter-observer agreement, the average of the precise TPS values obtained after the two assessments of each pathologist were computed and reclassified into the three large categories (<1, 1–49 and ≥50%) for each pathologist. We observed 15% of discordant cases between pathologists 1 and 2 for which the average TPS values were in the 1–49% category for one pathologist and <1% for the other (data not shown). The weighted κ index characterizing these data was 0.814 (CI, 0.709–0.918). Fig. 4 shows the scatterplot of the precise TPS averages obtained for the two pathologists, including numerous overlaps in the case of small values (see figure legend for details). The CCC computed on these TPS averages was 0.967 (CI, 0.950–0.978). These findings revealed that the 1–49% category was the least reproducible from one assessment to another, without evidence of an impact from sampling type (Fig. 4). Evaluation from a third pathologist allowed the completion of these inter-observer data (Fig. 5). For the three TPS categories, the weighted κ index was 0.722 (CI, 0.599–0.844) between pathologist 3 and 2 and 0.693 (CI, 0.566–0.820) between pathologist 3 and 1. The CCC computed on the precise TPS (Fig. 5) between pathologist 3 and 1 or 2 was 0.942 (CI, 0.912–0.963) and 0.943 (CI, 0.914–0.962), respectively. For pathologist 1 and 2, the latter results were obtained based on the TPS averages calculated from their two assessments (possibly smoothing out some variations). Fig. 5 details all the intra- and inter-observer variations by showing the precise TPS values (linked per case) provided by the three pathologists with two assessments for two of them. It should be noted that 24 perfect agreements of the 5 assessments on the TPS value of 0 were observed, resulting in overlapping horizontal lines at level 0.

## Discussion

Since PD-L1 expression is heterogeneous, dynamic and difficult to interpret, the present study aimed to identify factors that may affect the daily routine evaluation of PD-L1 expression from patients with NSCLC.

Regarding the pathological factors, the current study demonstrated that the sample location was significantly associated with PD-L1 expression. In particular, analysis of matched specimens from the same patient demonstrated a poor agreement between primary tumors and LN or distant metastasis. The results demonstrated that 43% patients with primary tumors not expressing PD-L1 had PD-L1-positive LN or metastasis, whereas 29% patients with PD-L1-positive primary tumors had PD-L1-negative LN or distant metastasis. This lack of agreement between the negative/positive PD-L1 status would have an impact on the possibility for patients to benefit from pembrolizumab. Only a few patients from the present study were treated with immunotherapy, which was a potential limitation of the present study. The results from the current study were similar to findings from Cho *et al* (25), who reported a 67% agreement between negative and positive PD-L1 expression on 91 matched samples of primary NSCLC and metastasis. In addition it was reported that 28% of the samples did not express PD-L1 after the first assessment but expressed PD-L1 after the second one (25). Conversely, 37% of PD-L1-positive samples became negative after the second assessment (25). In contrast, the ATLANTIC study reported an 89% agreement on 88 samples matched between primary tumors and metastasis (commercially available tissue samples); however, the result concerned PD-L1 expression rates of 25% and more (29). The data from Cho *et al* (25) and the present study suggested that these investigations should be performed on a larger sample size of matched samples from patients with clinical information regarding IT response in order to obtain more reliable data.

The present study also demonstrated that PD-L1 was more frequently expressed in SCC tissues compared with that in other histological types, and that its expression rate was mostly between 1 and 49%. Conversely, when ADCs and NSCLC NOS cases expressed PD-L1, the TPS score was higher. The SCC type has been proposed as a response factor to IT, as well as the smoking habit and the mutation number (3,30). These three variables may therefore be interconnected. SCC is more frequent in smokers, since tobacco, by increasing the mutation rate, produces neo-antigens that stimulate the immune response (3,5,31,32). SCC tumors may therefore be more sensitive to IT.

Regarding the specific mutations, a previous study on patients with EGFR-mutant NSCLC reported a significant association between PD-L1 positivity and *EGFR* mutations other than the L858R mutation or exon 19 deletion (33). The present study did not detect this association, as only 26 patients presented with *EGFR* mutations, including seven mutations other than the L858R mutation or exon 19 deletion. However, the present study demonstrated that *KRAS* mutations were associated with PD-L1 expression score. Patients with NSCLC and presenting with *KRAS* mutations express PD-L1 more frequently (64% (63/98) of KRAS mutated samples and 47% of KRAS non-mutated samples showed a TPS values ≥1%), as similarly observed by Li *et al* (34).

As reported by previous studies (10,15,35), the present study reported no significant difference in the PD-L1 expression rate between the different sampling types (cytology, biopsy or surgical specimen) in all patients combined. This result was confirmed by very good agreements between sample pairs from the same patient, as similarly described by Cho *et al* (25). Comparison of the present results with results from the Blueprint Phase 2B study, which compares the PD-L1 status between various samples types from the same lung tumor (16), is not yet possible.

The difficulty in correctly evaluating PD-L1 expression by IHC induces post-analytical heterogeneity, leading to intra- and inter-observer discrepancies. The concordance data from the present study were similar to those from the DREAM study (11,22). Regarding PD-L1 TPS assessment in three categories, the present study reported intra-observer discrepancies between 16 and 17.5%, suggesting a hypothesis that, in daily practice, 1–2 out of 10 patients were potentially misdiagnosed. Inter-observer discrepancies were not correlated with one particular sampling type. However, the most discordant cases were for patients with a pleural effusion sample, which is the most difficult type of sample to assess.

One current marker of interest is the Tumor Mutation Burden (TMB), which is defined as the total number of nonsynonymous mutations per coding area of a tumor genome. Although TMB is associated with IT effectiveness, it is not associated with PD-L1 expression (2,10,30–32). At present in our daily clinical practice, a NGS panel of 22 genes is used to search for ‘actionable’ mutations. The present study shows that this panel, which is smaller than the panels used for TMB, established a significant association between mutation number and PD-L1 expression, with a major difference between non-mutated NSCLC cases and NSCLC cases with >2 mutations.

In conclusion, to the best of our knowledge, the present study was one of the first investigating the impact of pathological and technological factors (such as tumor location and sampling type, respectively) in the daily clinical assessment of PD-L1 expression. The current study confirmed that cytological samples, which are often used routinely, can be used for the evaluation of PD-L1. The results from the present study also demonstrated significant associations between PD-L1 expression and sample location, histology, total number of mutations and KRAS mutations. These data will require further confirmation using a larger sample size. The results from the present study suggested that PD-L1 may be considered as a useful, although dynamic and heterogeneous, marker that may be associated with other clinicopathological characteristics of patients with NSCLC. Further investigation would improve its theranostic value.

## Figures and Tables

**Table I.** Clinicopathological characteristics of the patients with NSCLC included in the present study.

Characteristics	Number
Median age (n=454), (range)	66 (35–91)
Sex (%) (n=454)	
Female, n	172 (37.9)
Male, n	282 (62.1)
Smoking history (%) (n=145)	
Yes, n	132 (91.0)
No, n	13 (9.0)
Histology (%) (n=432)	
SCC, n	112 (25.9)
ADC, n	290 (67.1)
NSCLC NOS, n	24 (5.6)
Other^a^, n	6 (1.4)
Sampling type (%) (n=416)	
Cytology, n	87 (20.9)
Biopsy, n	235 (56.5)
Surgical specimen, n	94 (22.6)
Sample location (%) (n=414)	
Primary tumor	260 (62.8)
LN	88 (21.3)
Distant metastasis	66 (15.9)
pT (%) (n=74)	
T1	43 (58.1)
T2	12 (16.2)
T3	14 (18.9)
T4	5 (6.8)
pN (%) (n=153)	
N0	50 (32.7)
N1–2	103 (67.3)
pM (%) (n=112)	
M0	42 (37.5)
M1	70 (62.5)
Stage (%) (n=112)	
1	19 (17.0)
2	16 (14.3)
3	7 (6.2)
4	70 (62.5)
FFPE block age (%) (n=453)	
<3 years	446 (98.5)
≥3 years	7 (1.5)
*EGFR* mutation (%) (n=294)	
Yes	26 (8.8)
No	268 (91.2)
*KRAS* mutation (%) (n=289)	
Yes	108 (37.4)
No	181 (62.6)
*TP53* mutation (%) (n=287)	
Yes	108 (37.6)
No	179 (62.4)
Other mutations (%) (n=289)	
Yes	54 (18.7)
No	235 (81.3)
Total number of mutations (%) (n=287)	
0	61 (21.3)
1	162 (56.4)
2	53 (18.5)
3	10 (3.5)
4	1 (0.3)

a Other histology includes large cells carcinomas, sarcomatoid carcinomas, and pleomorphic carcinomas. pT, pN, pM and stage were revised according to the 8th UICC edition (26). ADC, adenocarcinoma; p, pathological; EGFR, epidermal growth factor receptor; FFPE, formalin-fixed paraffin-embedded; KRAS, KRAS proto-oncogene, GTPase; LN, lymph node metastasis; NOS, non-otherwise specified; SCC, squamous cell carcinoma; TP53, tumor protein p53.

**Table II.** Localization of distant metastasis.

Tissue/organ	Number (n=66)
Pleura	13
Brain	10
Liver	9
Pleural fluid	5
Bone	5
Adrenal glands	5
Cerebellum	3
Vertebra	3
Muscle	2
Diaphragm	2
Skin	2
Controlateral lung	1
Pericardial fluid	1
Mesentery	1
Pericardium	1
Pancreas	1
Subcutaneous	1
Paravertebra	1

**Table III.** Significant variations observed in PD-L1 TPS in relation to the clinicopathological characteristics described in Table I.

	PD-L1 TPS (3 categories)				Precise TPS value^a^	Precise TPS value ≥1%^a^
			
Variable	<1%, n (%)	1–49%, n (%)	≥50%, n (%)	P-value^b^	P-value^c,d^	P-value^c,d^
Sample location (n=414)						(n=241)
Primary	107 (41)	90 (35)	63 (24)	0.005	NS^d^	0.020^d^
LN	42 (48)	13 (15)	33 (38)			
Metastasis	24 (36)	19 (29)	23 (35)			
Stage^e^ (n=42)						(n=27)
1–2	9 (29)	13 (42)	9 (29)	NA	0.049^c^	NA^c^
3–4	6 (55)	4 (36)	1 (9)			
Histology (n=426)^f^						(n=247)
SCC	37 (33)	47 (42)	28 (25)	0.026	NS^e^	0.036^e^
ADC	129 (44)	79 (27)	82 (28)			
NSCLC NOS	13 (54)	4 (17)	7 (29)			
Total number of mutations (n=287)						(n=158)
0	37 (61)	10 (16)	14 (23)	0.004	0.002^d^	NS^d^
1	70 (43)	50 (31)	42 (26)			
>1	22 (34)	14 (22)	28 (44)			
*KRAS* mutations (n=256)^g^						(n=138)
Yes	35 (36)	28 (29)	35 (36)	0.024	0.0006^c^	NS^c^
No	83 (53)	38 (24)	37 (23)			

a The column ‘precise TPS value’ refers to the detailed assessment in 13 categories (see Methods) and takes into account all samples. The column ‘precise TPS value ≥1%’ excludes all the cases with a precise TPS value of 0. The resulting number of cases are detailed for each characteristic.

b χ^2^ test.

c Mann-Whitney or

d Kruskal-Wallis test for comparing 2 or >2 independent data groups, respectively.

e Considering primary tumors only (n=42).

f Considered only SCC, ADC and NSCLC NOS because of too few cases in the ‘other’ category for statistical analysis.

g Considered only the ADC and NSCLC NOS in this analysis (because of the insufficient molecular data available for the other histological types), resulting in 256 cases analyzed. ADC, adenocarcinoma; KRAS, KRAS proto-oncogene, GTPase; LN, lymph node metastasis; NA, not applicable; NOS, non-otherwise specified; NS, not significant; SCC, squamous cell carcinoma; TPS, Tumor Proportion Score; PD-L1, PD-L1 molecule.

**Table IV.** Breakdown of matched data characterizing PD-L1 TPS into three categories in primary tumor, LN or distant metastasis samples from the same patient.

	LN/Metastasis TPS, n			
		
Primary tumor TPS	<1%	1–49%	≥50%	Total, n
<1%	8	3	3	14
1–49%	3	1	0	4
≥50%	1	7	2	10
Total, n	12	11	5	28

LN, lymph node metastasis; TPS, tumor proportion score.

**Table V.** Intra-observer agreement of the PD-L1 molecule TPS assessments into three categories by pathologist 1 (junior) and pathologist 2 (senior).

A, Pathologist 1-WKI: 0.779 (CI, 0.665–0.893)				

	2nd TPS assessment, n			
		
1st TPS assessment, n	<1%	1–49%	≥50%	Total, n
<1%	38	4	0	42
1–49%	9	14	1	24
≥50%	0	0	14	14
Total, n	47	18	15	80

**B, Pathologist 2-WKI: 0.804 (CI, 0.703–0.905)**				

	**2nd TPS assessment, n**			
		
**1st TPS assessment, n**	**<1%**	**1–49%**	**≥50%**	**Total**

<1%	40	2	0	42
1–49%	7	12	2	21
≥50%	0	2	15	17
Total, n	47	16	17	80

WKI, weighted κ index; TPS, tumor proportion score.

## Data Availability

The datasets used and/or analyzed during the current study are available from the first author on reasonable request.

## Abbreviations

ADC: adenocarcinoma
IHC: immunohistochemistry
IT: immunotherapy
KRAS: KRAS proto-oncogene
LN: lymph node metastasis
NGS: next generation sequencing
NSCLC: non-small cell lung cancer
PD-L1: programmed death-ligand 1
PDCD-1: programmed cell death 1
SCC: squamous cell carcinoma
TPS: tumor proportion score
